# Serological prevalence of SARS-CoV-2 infection and associated factors in healthcare workers in a “non-COVID” hospital in Mexico City

**DOI:** 10.1371/journal.pone.0255916

**Published:** 2021-08-12

**Authors:** Esteban Cruz-Arenas, Elizabeth Cabrera-Ruiz, Sara Laguna-Barcenas, Claudia A. Colin-Castro, Tatiana Chavez, Rafael Franco-Cendejas, Clemente Ibarra, Javier Perez-Orive

**Affiliations:** 1 Epidemiological Vigilance Unit, Instituto Nacional de Rehabilitacion “Luis Guillermo Ibarra Ibarra”, Mexico City, Mexico; 2 Instituto Nacional de Rehabilitacion “Luis Guillermo Ibarra Ibarra”, Mexico City, Mexico; 3 Infectious Diseases Division, Instituto Nacional de Rehabilitacion “Luis Guillermo Ibarra Ibarra”, Mexico City, Mexico; Medicina Fetal Mexico, MEXICO

## Abstract

**Background:**

Mexico is one of the countries with the highest number of deaths from the COVID-19 pandemic. In spite of this high mortality, in Mexico the number of confirmed cases and diagnostic tests per million population are lower than for other comparable countries, which leads to uncertainty about the actual extent of the pandemic. In Mexico City, healthcare workers represent an important fraction of individuals with SARS-CoV-2 infection. We performed a cross-sectional study whose objective was to estimate the frequency of antibodies to SARS-CoV-2 and identify associated factors in healthcare workers at a large hospital in Mexico City.

**Methods:**

We conducted a serological survey in a non-COVID national referral teaching hospital. The study population included all the personnel that works, in any capacity, in the hospital. From this population we selected a representative sample of 300 individuals. Blood samples were collected and questionnaires were applied between August 10^th^ and September 9^th^, 2020.

**Results:**

ELISA results indicate a serological prevalence of SARS-CoV-2 infection of 13.0%. Working in the janitorial and security groups, having an educational level below a university degree, and living with a larger number of people, were all identified as sociodemographic factors that increase the probability of having SARS-CoV-2 infection.

**Conclusions:**

Less favored socioeconomic groups face significantly higher prospects of experiencing SARS-CoV-2 infection and in institutions such as ours, providing janitorial and security workgroups with additional testing and counseling could help to limit the spread of contagion. The rate from the official number of confirmed cases in Mexico City is substantially smaller than the seropositive rate identified in this work.

## Introduction

Severe acute respiratory syndrome coronavirus 2 (SARS-CoV-2) is the cause of Coronavirus disease 2019 (COVID-19). As of October 30^th^, 2020, countries in the Americas occupy three of the first four places in number of deaths: the United States of America (USA) with 230,159, Brazil with 158,456 and Mexico with 90,309 [1]. However, the number of reported cases per million inhabitants in Mexico (7,034) is much lower than that of the USA and Brazil (with 26,672 and 25,726, respectively) [1]. The number and criteria for conducting real time polymerase chain reaction (RT-PCR) tests in each country have also been different. As of October 30^th^ 2020, the estimates are 16.1 tests per million inhabitants for Mexico, compared to 456.0 for the United States and 30.2 for Brazil [2]. This smaller number of RT-PCR tests in Mexico is related to a very high positivity rate of tests that was 41.3% on September 9^th^ 2020 [3]. This leads to much uncertainty about the actual number of COVID-19 infections that have occurred in countries with low numbers of tests per million population. Mexican government measures to attend the health emergency included the conversion of hospitals into exclusive units for the management of pandemic patients, i.e. "COVID hospitals", and "non-COVID hospitals” which would focus on non-COVID-19 patients requiring other urgent medical care; designation of the sentinel model of epidemiological surveillance [1], and focusing RT-PCR tests on people with symptoms [3]. Healthcare workers (HCW) represent an important fraction of individuals with SARS-CoV-2 infection in Mexico City [4]. Accurate epidemiological information is required to support public health actions aimed at limiting contagion and loss of life, while minimizing negative economic impact to the population [1]. With this purpose, serological surveys estimating SARS-CoV-2 infection have been conducted in many places globally [5–15]. In Mexico there is a lack of seroprevalence studies that allow for a more accurate description of the prevalence of SARS-CoV-2 infections in different Mexican populations [16].

This work provides serological data from a non-COVID national referral teaching hospital, its main objective being to estimate the frequency of antibodies to SARS-CoV-2 in HCW at a large non-COVID hospital in Mexico City. By conducting a structured questionnaire with the study participants, it also allows us to identify sociodemographic and other factors associated with SARS-CoV-2 infections.

## Materials and methods

### Study population

We performed a cross-sectional study, having as study population the personnel that works in our hospital, the Instituto Nacional de Rehabilitación “Luis Guillermo Ibarra Ibarra”, in Mexico City (n = 2974). Our institution is a public national referral teaching hospital with 235 beds located in Mexico City and was designated by the federal health authorities as a non-COVID hospital. Hospitals designated as non-COVID delayed elective surgeries focusing on urgencies. Our institution became one of the few public hospitals in Mexico City attending trauma patients. Upon arrival at the emergency room, all patients and relatives were asked about possible COVID-19 contacts, respiratory symptoms and comorbidities in order to identify infection risk factors. During their stay at the emergency room patients were clinically evaluated and then discharged to the next filter where they were swabbed for the RT-PCR test. Non-COVID hospitals are not prepared to treat COVID-19 patients, thus if any patient was identified as infected he or she was referred to a COVID hospital if their symptoms required hospital attention, otherwise they were directed to self-isolate at home and asked to come back once the infection subsided [3]. To evaluate factors associated with SARS-CoV-2 infection, we verbally applied once a structured questionnaire to the study population. Serological status was evaluated with Lateral Flow Assay (LFA) and Enzyme-Linked Immunosorbent Assay (ELISA) tests applied to peripheral blood samples. We used a sample size of 300 subjects (sample size calculation described in the statistical analysis section). The subjects in the sample were stratified in 10 groups according to their work activities: administrative; scientific research; medical personnel; nursing; stretcher-bearers and orderlies; technicians and laboratory personnel; therapists and patient counseling; janitorial; security; and food services. We selected participants randomly from each of the 10 work group strata, according to the size of each work group (S1 Fig). Inclusion criteria were: being personnel working in the hospital, from any shift, and granting written informed consent. Thus, all hospital personnel had the same probability of being included in the study, avoiding selection bias. Participants were contacted by telephone, those that did not consent to participate in the study were replaced by other participants selected by the same randomized process. The study was approved by the Institute’s Ethics and Research Committees (*Comité de Ética en Investigación* and *Comité de Investigación*, both of the Instituto Nacional de Rehabilitación “Luis Guillermo Ibarra Ibarra”; approval number INR-26/20, approval date July 6^th^, 2020) and was carried out in accordance with relevant guidelines and regulations, including the Declaration of Helsinki. Written informed consent was obtained from all participants. We scheduled study participants between August 10^th^ and September 9^th^, 2020, to obtain a peripheral blood sample and complete a structured questionnaire. We conducted procedures in a specially designated area, following World Health Organization safety guidelines with both participants and study personnel, including use of facemasks, physical distancing, ventilated area, and hand hygiene. The measurement of all study variables were obtained under the same conditions for all participants (regardless of their positive or negative outcome), minimizing the probability of information bias.

### Definition of variables

In the structured questionnaire, we explored the following exposure variables associated with SARS-CoV-2 antibodies: sociodemographic variables, COVID-19 previous or present signs and symptoms, diagnostic RT-PCR tests, comorbidities, status of seasonal influenza vaccine, living arrangements (including number of people sharing a home), use of public transportation for work commute, degree of exposure to patients, use of personal protective equipment, number of meals per day, physical activity, and hours of sleep. To determine the outcome variable we conducted two serological tests on peripheral blood samples, an LFA and an ELISA. The LFA tests were the COVID-19 IgG/IgM Rapid Test Cassette® (Hangzhou Biotest Biotech Co. Ltd., China), which use an immunochromatographic assay to provide qualitative detection of IgM and IgG antibodies against the SARS-CoV-2 virus. We considered a participant to be positive with the LFA test if they had a positive result for either IgG, IgM or both. The ELISA tests we used were the Euroimmun® Anti-SARS-CoV-2 NCP which detect IgG antibodies against the SARS-CoV-2 nucleocapsid protein (Euroimmun Medizinische Labordiagnostika, AG, Germany). According to the manufacturer’s guidelines, a positive result is one with a resulting ratio ≥ 1.1, negative ≤ 0.8 and the interval between these values is considered uncertain. Thus, the result of the ELISA test was considered a dichotomous variable: positive or negative for SARS-CoV-2 antibodies, with the uncertain values (which were only three from the total study population) not included in the final regression analysis. Educational level, number of people with which participants live, having family members positive for COVID-19 and contact with patients at work were considered as potential confounding variables.

### Statistical analysis

We conducted all statistical analyses with Stata v13.0 (StataCorp, USA). The sample size was calculated with the objective of determining prevalence of antibodies to SARS-CoV-2 in our population. We assumed a positivity rate of 10%, and a significance level of 5%, with which we calculated a sample size of 139. In order to increase the sample power, and given the lack of information about COVID-19 prevalence in the Mexican population, we selected a final sample size of 300 subjects (S1 Fig). We report proportions of sample study groups and qualitative variables as percentages. We used multiple logistic regression analysis to obtain odds ratios (OR), 95% confidence intervals (CI), and *p* values, in order to evaluate associations between the variables and the categorical results of the ELISA tests. All those associations with *p* values ≤ 0.25 were selected to adjust models using multiple logistic regression. Each variable was then incorporated into the model and only those that had *p* ≤ 0.05 were retained. To assess the adjustment of the variables included in the final model, we report pseudo R^2^ values. Variables considered potentially confounding were incorporated again into the final model to determine their effect. With the exceptions of age, hours of sleep, and number of people with which participants live, which were considered as quantitative variables, all other characteristics in the analysis were considered as categorical variables. Finally, and using the thresholds established by the ELISA test manufacturer, we calculated the sensitivity, specificity, positive and negative predictive values of the LFA test.

## Results

### Study population

The sample included a total of 300 participants, all of whom responded to structured questionnaires and had LFA tests applied. ELISA tests were conducted on 299 participants, since in one subject it was not possible to extract enough blood volume for the ELISA test, but only for the LFA test (S1 Fig). As observed in Table 1, the largest work group strata were administrative (21.4%), medical personnel (19.4%), and nursing (18.7%). The majority of participants work in the morning shift (60.2%), are female (65.2%; in line with the overall population working in the hospital), single (49.8%), belong to the 30–44 age group (41.5%), and reported having a university bachelor’s degree or higher (72.9%), as well as high levels of patient contact (69.6%).

**Table 1:** General characteristics of the study population in relationship to the categorical ELISA test results.

Variable	Sample size	Proportion of sample, %	No. positive with ELISA (IgG)	Proportion of positive for IgG, %
**Total sample with ELISA test**	**299**	**100**	**39**	**13.0**
Work group strata				
Administrative	64	21.4	8	12.5
Scientific research	16	5.3	1	6.2
Medical personnel	58	19.4	3	5.2
Nursing	56	18.7	7	12.5
Stretcher-bearers and orderlies	16	5.3	1	6.2
Technicians and lab personnel	18	6.0	2	11.1
Therapists and patient counseling	47	15.7	6	12.8
Janitorial	11	3.7	5	45.4
Security	8	2.7	5	62.5
Food services	5	1.7	1	20.0
Sex				
Male	104	34.8	9	8.7
Female	195	65.2	30	15.4
Age (years)				
< 30	41	13.7	7	17.1
30–44	124	41.5	19	15.3
45–59	114	38.1	11	9.6
≥ 60	20	6.7	2	10.0
Marital status				
Single	149	49.8	17	11.4
Married or civil union	143	47.8	20	14.0
Widow/widower	7	2.4	2	28.6
Educational level				
Less than University degree	81	27.1	19	23.5
University bachelor’s degree or higher	218	72.9	20	9.2
Shift				
Morning	180	60.2	25	14.0
Evening	57	19.1	9	15.8
Night	21	7.0	2	9.5
Other	41	13.7	3	7.3
Contact with patients during work				
No or limited contact	91	30.4	14	15.4
High contact	208	69.6	25	12.0
LFA				
Positive to IgM and IgG	13	4.3	13	100
Positive to IgM only	2	0.7	0	0.0
Positive to IgG only	18	6.0	18	100
Negative	266	89.0	8	3.0
RT-PCR				
Positive	10	3.3	8	80.0
Negative	77	25.7	5	6.5
Not tested	212	71.0	26	12.3
Having presented symptoms or suspected having COVID-19				
Yes	138	46.1	20	14.5
No	161	53.9	19	11.8

LFA: Lateral Flow Assay test.

RT-PCR: Real Time Polymerase Chain Reaction.

### Prevalence of SARS-CoV-2 antibodies

The LFA tests resulted in a prevalence of SARS-CoV-2 antibodies of 11.0% (n = 33). Of these, 13 (39.4%) presented both IgM and IgG, 18 (54.5%) only IgG, and 2 (6.1%) only IgM. For the ELISA test, which measures only IgG and not IgM, and considering the result as a categorical variable according to the manufacturer’s threshold for positivity described in the methods, the serological prevalence was 13.0% (n = 39), slightly higher than with the LFA test (Table 1). Based on the contacts reported by participants with positive results for the ELISA test (n = 39), we estimate that 13 cases (33.3%) were likely to be infected outside the hospital, 14 cases (35.9%) inside, and in 12 cases (30.8%) there is insufficient information to estimate. Of these 39 positive subjects, 7 individuals (17.9%) reported working additional shifts in other hospitals, 3 of them (7.7%) in a COVID hospital, and 4 (10.3%) in other non-COVID hospitals. Within these same positive subjects, close to half (n = 19, 48.7%) reported the day of the blood sample not having experienced related symptoms, nor suspected having had COVID-19 (Table 1).

### Test comparisons

From our sample of 300 participants, 87 (29.0%) reported they had previously had RT-PCR tests for SARS-CoV-2, 10 of them having positive results (11.5% positivity rate). The median number of days between the date of the RT-PCR tests that were positive and when the blood sample was taken in these 10 individuals was 93 days (range, 48–117 days). The concordance between the 10 RT-PCR positive subjects and the ELISA test was 8 (80.0%), slightly higher than with the LFA test which was 7 (70.0%). Compared to the ELISA test, the LFA test had sensitivity of 79.5%, specificity of 100.0%, positive predictive value of 100.0% and negative predictive value of 97.0%.

### Factors associated with SARS-CoV-2 infection

Remarkably, the security and janitorial work groups had substantially higher rates of positive results (62.5% and 45.4%, respectively) than the other work groups (Table 1). Additionally, participants with educational level below university degree also had higher rates of positive results than those with a higher educational level (23.5% vs. 9.2%). In the bivariate analysis, the variables that presented ORs with the strongest associative strength to the level of anti-SARS-CoV-2 IgG antibodies are: working in the security and janitorial groups, lower educational level, larger number of persons with which participants live, and the following symptoms: muscle and joint pain, dyspnea, fever, olfactory alterations and dysgeusia (Table 2).

**Table 2:** Logistic regression analysis of associations between variables and ELISA test results.

Variable	Unadjusted OR	95% CI		*p*
		Lower	Upper	
Work group strata				
Administrative	Ref.	-	-	-
Scientific research	0.46	0.05	3.96	0.478
Medical personnel	0.37	0.09	1.49	0.163
Nursing	1.00	0.34	2.97	0.997
Stretcher-bearers and orderlies	0.49	0.06	4.26	0.519
Technicians and lab personnel	0.86	0.16	4.46	0.857
Therapists and patient counseling	1.01	0.32	3.12	0.992
Janitorial	5.73	1.41	23.22	0.014
Security	11.46	2.28	57.44	0.003
Food services	1.72	0.17	17.37	0.646
Sex (male)	0.52	0.24	1.14	0.104
Age (years)	0.98	0.95	1.01	0.240
Educational level (University bachelor’s degree or higher)	0.33	0.16	0.65	0.002
Symptoms between March 2020 and blood sample date					
Muscle and joint pain	3.20	1.55	6.60	0.002
Headache	1.43	0.71	2.88	0.311
Cough	1.75	0.74	4.13	0.201
Odynophagia	1.07	0.46	2.46	0.877
Rhinorrhea	1.49	0.63	3.48	0.360
Dyspnea	2.73	1.00	7.49	0.050
Fever	3.23	1.30	8.00	0.011
Diarrhea	1.78	0.78	4.06	0.168
Conjunctivitis	1.94	0.73	5.14	0.181
Nausea	0.42	0.05	3.31	0.413
Shivering	1.45	0.40	5.28	0.576
Olfactory alterations	17.50	7.44	41.17	<0.001
Dysgeusia	21.29	8.50	53.33	<0.001
Presence of comorbidities	0.64	0.30	1.38	0.260
Status of seasonal influenza vaccine	1.03	0.52	2.04	0.941
Previous COVID-19 cases in family	1.43	0.71	2.88	0.311
Number of people with which participants live	1.19	1.02	1.39	0.029
Living with people who worked outside home during the pandemic	2.19	0.42	11.28	0.349
Background of having cared for people with COVID-19	0.96	0.46	2.00	0.925
Using public transportation for work commute	1.62	0.82	3.21	0.167
Contact with patients at work (high contact)	0.75	0.37	1.52	0.425
Familiarity and use of personal protection equipment	1.19	0.55	2.57	0.649
Number of meals per day (< 3)	1.43	0.69	2.96	0.330
Being physically active	0.70	0.36	1.39	0.311
Hours of sleep	1.00	0.76	1.30	1.000

Table 3 shows the multiple logistic regression model using the results (positive or negative) of the ELISA test, and retaining only those variables with a p < 0.05 (see Methods). It shows that male participants had lower probability of presenting IgG antibodies than female participants (OR = 0.31, 95% CI, 0.11–0.90). Reporting olfactory alterations was significantly associated with higher probability of presenting antibodies (OR = 34.73, 95% CI, 11.41–105.68). The model shows additionally that the janitorial (OR = 13.70, 95% CI, 2.85–65.77) and security (OR = 12.35, 95% CI, 1.32–115.01) work groups were strongly associated with the ELISA results. Once this final model was obtained, to assess the effect of potential confounding variables we incorporated them into the model (see Methods) and determined that only the educational level exhibits characteristics of a confounding variable, since in the model that includes it the relationship between antibody presence and working in the security work group ceases to be significant (S1 Table). The rest of the potential confounding variables explored explain independently the presence of antibodies among the study participants (S2–S4 Tables).

**Table 3:** Logistic regression model between associated variables and ELISA test results.

Variable	Adjusted	95% CI		*p*
	OR	Lower	Upper	
Sex (male)	0.31	0.11	0.90	0.031
Olfactory alterations	34.73	11.41	105.68	<0.001
Work group strata				
Administrative	Ref.	-	-	-
Scientific research	1.27	0.13	12.23	0.838
Medical personnel	0.32	0.06	1.66	0.175
Nursing	0.71	0.18	2.78	0.626
Stretcher-bearers and orderlies	0.77	0.07	8.41	0.834
Technicians and lab personnel	0.73	0.09	5.60	0.761
Therapists and patient counseling	1.81	0.48	6.83	0.379
Janitorial	13.70	2.85	65.77	0.001
Security	12.35	1.32	115.01	0.027
Food services	5.58	0.47	66.31	0.174
	**Pseudo *R*** ^***2***^ **= 0.32**			

## Discussion

In this work we provide results from a seroprevalence survey in a large non-COVID teaching hospital in Mexico City. The ELISA results indicate that 13.0% of the sample are positive to IgG antibodies to SARS-CoV-2. Our study also identified sociodemographic factors, all of which are linked to less favored socioeconomic groups, associated with previous infection with SARS-CoV-2: working in the janitorial and security groups, having an education level below university bachelor’s degree, and living with a larger number of people.

### Seroprevalence

Compared with studies measuring seropositivity for HCW in other countries, the antibody prevalence observed in our study for the whole hospital (13.0%) is within the range of that found for other countries: 19.1% for a large hospital in Sweden [12], 10.2% for nationwide HCW in Spain [8] and 6.2% for a tertiary care center in Belgium [15]. Since our hospital was designated non-COVID, it is to be expected to have fewer infections than in a hospital that was focused on COVID-19 patients. Additionally, there is still patient contact in our hospital, and some patients that later were known to have positive RT-PCR tests were treated by the subjects in our sample. Thus, in a sense it is more representative of the general population, as well as other work environments where SARS-CoV-2 positive individuals occur randomly, rather than being specifically grouped as in a COVID-19 hospital.

A study with HCW in London found that 21.9% of seropositive subjects did not report COVID-19 symptoms [13]. In this work, almost half (46.3%) of seropositive subjects had experienced no symptoms and did not suspect they had previously had SARS-CoV-2 infection. This large percentage of subjects not suspecting they had previously had COVD-19 underscores the importance of seroprevalence surveys including asymptomatic subjects as fundamental in order to obtain accurate information regarding the extent of infections in a population [16, 17].

Recent studies have found that seropositivity of antibodies to SARS-CoV-2 decreases substantially over a 60-day period of time [18]. Additionally, it is unclear whether all patients with COVID-19 elevate antibodies, and in which time frame [19–22]. This implies that in our current study we may not be capturing all the subjects that might have been previously infected with SARS-CoV-2, and therefore our result of 13.0% prevalence of SARS-CoV-2 should be considered as a lower bound in terms of the prevalence of previous infection to this virus. Another point that must be considered is that given that the questionnaire and blood samples were taken the same day, we do not have precise knowledge of the temporal sequence of events, and therefore a longitudinal study would provide more clarity in this regard.

### Associated factors

Our logistic regression model based on the ELISA test indicates that male subjects tend to have lower antibody scores than female subjects. This is in line with previous studies finding that in some groups of COVID-19 patients the generation of IgG antibodies is stronger in women than men [23] and that in general women mount stronger immune responses to infections and vaccinations [24].

In addition to physiological factors that are known to be associated with COVID-19, such as olfactory alterations, fever, muscle and joint aches, our logistic regression analysis (Table 2) finds that the prevalence of antibodies is significantly associated with three sociodemographic factors: number of people with which one lives, having an educational level below a university bachelor’s degree, and type of work, with subjects working as janitors and security guards having substantially higher probability of past infection than other personnel groups such as physicians, nurses and administrative staff. In the logistic regression model, educational level exhibits characteristics of a confounding variable with working in the security group. Taken together, our findings suggest that in this population, less favored socioeconomic groups face significantly higher probability of being infected with SARS-CoV-2. Interestingly, another seroprevalence study among HCW from the New York City area [14] also found that service/maintenance personnel (including, housekeepers and groundkeepers, among others) had a substantially larger level of SARS-CoV-2 exposure (20.9%) than other professions such as physicians (8.7%) and allied health professionals such as physical and occupational therapists (11.6%). This suggests that the sociodemographic factors identified in our seroprevalence survey may also be associated with higher SARS-CoV-2 exposure in other contexts, including other countries.

## Limitations and concluding remarks

The random selection process in which all personnel of our hospital had the same chance of being included minimizes selection bias. As mentioned above, a limitation inherent to a cross-sectional study design is not being able to determine the temporal relationship between the associations. Also, as in all cross-sectional observational studies, the impossibility of establishing causal relationships between exposure and outcome variables must be kept in mind.

Our sample can be representative of other non-COVID hospitals with similar work group structure as ours in other regions in Mexico with similar degrees of contagion to Mexico City. Additionally, given the filters limiting access to the hospital to COVID-19 patients, in the absence of other data it might also provide relevant information to other non-healthcare institutions or organizations with similar sociodemographic and educational breakups as our institution. Seroprevalence studies in various populations can be a tool to provide useful information for planning public health measures at institutional, regional and national levels. For instance, our findings can help guide public health policies at the institutional level, such as identifying specific populations facing higher probability of SARS-CoV-2 infection (which can therefore be potential sources of further contagion in the workplace) and focusing attention on these groups by providing additional testing as well as informative sessions and counseling regarding preventive COVID-19 measures. By September 9^th^ 2020, which is the date our last blood sample was collected, the number of confirmed cases in Mexico City was 107,613 [3] which represented 1.2% of the city’s population. Given the sentinel model of epidemiological surveillance that has been followed by the Mexican authorities, the more than ten-fold difference between this number and the 13.0% obtained in our study is not surprising and cannot be accounted for as a difference caused by factors that are specific to HCWs. Although not representative at a regional or national level, in the current absence of other data, this seroprevalence survey provides useful information that can give a sense of the extent of the pandemic in a specific segment of the Mexican population and contribute valuable information to guiding public health policies.

Finally, our study adds data suggesting that less favored socioeconomic groups face significantly higher prospects of experiencing SARS-CoV-2 infection. In institutions such as ours, providing janitorial and security workgroups with additional testing and counseling could help to limit the spread of contagion.

## Supporting information

S1 Fig Study population and randomization process flow chart. (TIF)

S1 Table Logistic regression model adjusted by confounding variable: Educational level. (PDF)

S2 Table Logistic regression model adjusted by confounding variable: Number of people with which participants live. (PDF)

S3 Table Logistic regression model adjusted by confounding variable: Previous COVID-19 cases in family. (PDF)

S4 Table Logistic regression model adjusted by confounding variable: Contact with patients at work (high contact). (PDF)

S1 Dataset Anonymized data set. (XLSX)

S1 File Spanish original language questionnaire. (PDF)

S2 File English translation questionnaire. (PDF)
