# Supplementary figures and images for: Serological prevalence of SARS-CoV-2 infection and associated factors in healthcare workers in a “non-COVID” hospital in Mexico City
Source: PLoS One. 2021 Aug 12;16(8):e0255916. doi: 10.1371/journal.pone.0255916 (PMC8360585; doi:10.1371/journal.pone.0255916)

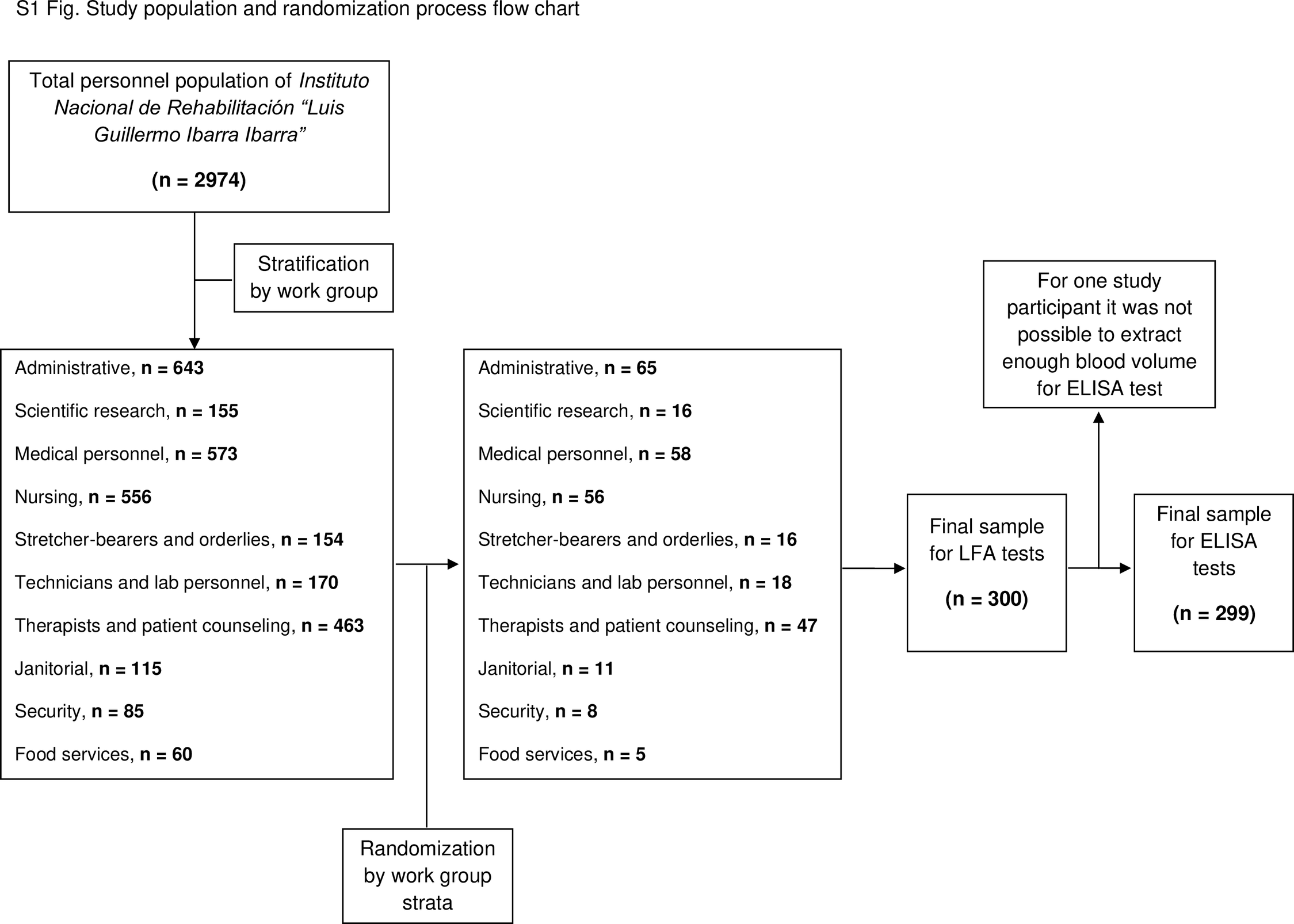

Supplement: S1 Fig — (TIF) [file pone.0255916.s001.tif]
