# Supplementary material for: Serological prevalence of SARS-CoV-2 infection and associated factors in healthcare workers in a “non-COVID” hospital in Mexico City
Source: PLoS One. 2021 Aug 12;16(8):e0255916. doi: 10.1371/journal.pone.0255916 (PMC8360585; doi:10.1371/journal.pone.0255916)
Supplement: S1 Table — (PDF) [file pone.0255916.s002.pdf]

S1 Table. Logistic regression model adjusted by confounding variable: educational level.

Logistic regression model between associated variables and result odds from ELISA tests. [a]				
**Variable**	**Adjusted OR**	**95% CI**		***P***
		**Lower**	**Upper**	
Sex (male)	0.32	0.11	0.93	0.036
Olfactory alterations	35.48	11.55	109.04	<0.001
Work group strata				
Administrative	Ref.	-	-	-
Scientific research	1.97	0.16	23.51	0.591
Medical personnel	0.49	0.07	3.27	0.463
Nursing	1.06	0.21	5.30	0.934
Stretcher-bearers and orderlies	0.61	0.05	7.12	0.696
Technicians and lab personnel	0.96	0.12	7.66	0.971
Therapists and patient counseling	2.69	0.55	13.16	0.221
Janitorial	10.44	2.01	54.08	0.005
Security	9.23	0.92	92.24	0.058
Food services	4.22	0.34	52.82	0.264
Educational level				
University bachelor’s degree or higher	0.49	0.12	2.05	0.328
		**Pseudo R^2 = 0.32**		

[a] Model adjusted by sex, olfactory alterations, work group strata and by one potentially confounding variable: educational level.
