# Supplementary material for: Serological prevalence of SARS-CoV-2 infection and associated factors in healthcare workers in a “non-COVID” hospital in Mexico City
Source: PLoS One. 2021 Aug 12;16(8):e0255916. doi: 10.1371/journal.pone.0255916 (PMC8360585; doi:10.1371/journal.pone.0255916)
Supplement: S2 Table — (PDF) [file pone.0255916.s003.pdf]

S2 Table. Logistic regression model adjusted by confounding variable: number of people with which participants live.

Logistic regression model between associated variables and result odds from ELISA tests. [a]				
**Variable**	**Adjusted OR**	**95% CI**		***P***
		**Lower**	**Upper**	
Sex (male)	0.32	0.11	0.92	0.035
Olfactory alterations	33.35	10.81	102.90	<0.001
Work group strata				
Administrative	Ref.	-	-	-
Scientific research	1.30	0.13	12.94	0.820
Medical personnel	0.35	0.06	1.91	0.228
Nursing	0.73	0.18	2.89	0.651
Stretcher-bearers and orderlies	0.73	0.07	7.74	0.793
Technicians and lab personnel	0.84	0.11	6.46	0.872
Therapists and patient counseling	2.00	0.52	7.73	0.315
Janitorial	13.77	2.80	67.65	0.001
Security	13.21	1.37	127.10	0.025
Food services	6.14	0.51	73.71	0.152
Number of people with which participants live	1.18	0.95	1.46	0.136
		**Pseudo R^2 = 0.33**		

[a] Model adjusted by sex, olfactory alterations, work group strata and one potentially confounding variable: number of people with which participants live.
