# Supplementary material for: Serological prevalence of SARS-CoV-2 infection and associated factors in healthcare workers in a “non-COVID” hospital in Mexico City
Source: PLoS One. 2021 Aug 12;16(8):e0255916. doi: 10.1371/journal.pone.0255916 (PMC8360585; doi:10.1371/journal.pone.0255916)
Supplement: S3 Table — (PDF) [file pone.0255916.s004.pdf]

S3 Table. Logistic regression model adjusted by confounding variable: previous COVID-19 cases in family.

Logistic regression model between associated variables and result odds from ELISA tests. [a]				
**Variable**	**Adjusted OR**	**95% CI**		***p***
		**Lower**	**Upper**	
Sex (male)	0.32	0.11	0.92	0.034
Olfactory alterations	33.42	10.89	102.54	<0.001
Work group strata				
Administrative	Ref.	-	-	-
Scientific research	1.24	0.13	11.95	0.852
Medical personnel	0.32	0.06	1.64	0.173
Nursing	0.68	0.17	2.69	0.587
Stretcher-bearers and orderlies	0.72	0.06	8.08	0.790
Technicians and lab personnel	0.71	0.09	5.61	0.747
Therapists and patient counseling	1,87	0.50	7.06	0.355
Janitorial	14.39	2.97	69.65	0.001
Security	12.35	1.33	114.40	0.027
Food services	5.98	0.50	71.93	0.159
Previous COVID-19 cases in family	1.29	0.50	3.31	0.159
		**Pseudo R^2 = 0.32**		

[a] Model adjusted by sex, olfactory alterations, work group strata and one potentially confounding variable: previous COVID-19 cases in family.
