# Supplementary material for: Serological prevalence of SARS-CoV-2 infection and associated factors in healthcare workers in a “non-COVID” hospital in Mexico City
Source: PLoS One. 2021 Aug 12;16(8):e0255916. doi: 10.1371/journal.pone.0255916 (PMC8360585; doi:10.1371/journal.pone.0255916)
Supplement: S4 Table — (PDF) [file pone.0255916.s005.pdf]

S4 Table. Logistic regression model adjusted by confounding variable: contact with patients at work (high contact).

Logistic regression model between associated variables and result odds from ELISA tests.[a]				
**Variable**	**Adjusted OR**	**95% CI**		***P***
		**Lower**	**Upper**	
Sex (male)	0.31	0.11	0.90	0.031
Olfactory alterations	35.13	11.54	106.92	<0.001
Work group strata				
Administrative	Ref.	-	-	-
Scientific research	1.24	0.13	12.03	0.849
Medical personnel	0.35	0.06	1.92	0.226
Nursing	0.81	0.19	3.49	0.775
Stretcher-bearers and orderlies	0.75	0.07	8.38	0.819
Technicians and lab personnel	0.76	0.10	5.83	0.791
Therapists and patient counseling	1,98	0.50	7.88	0.332
Janitorial	13.51	2.82	64.77	0.001
Security	12.49	1.34	116.63	0.027
Food services	5.21	0.43	63.23	0.195
Contact with patients at work (high contact)	0.79	0.29	2.17	0.653
		**Pseudo R^2 = 0.32**		

[a] Model adjusted by sex, olfactory alterations, work group strata and one potentially confounding variable: contact with patients at work (high contact).
