# Supplementary material for: Serological prevalence of SARS-CoV-2 infection and associated factors in healthcare workers in a “non-COVID” hospital in Mexico City
Source: PLoS One. 2021 Aug 12;16(8):e0255916. doi: 10.1371/journal.pone.0255916 (PMC8360585; doi:10.1371/journal.pone.0255916)
Supplement: S1 File — (PDF) [file pone.0255916.s007.pdf]

Cuestionario para el estudio: Serological prevalence of SARS-CoV-2 infection and associated factors in healthcare workers in a “non-COVID” hospital in Mexico City.
Tipo de cuestionario: Directo, entrevista cara-cara.

1. Grupo de trabajo asignado:

- Administrativos
- Investigadores
- Personal médico
- Enfermería
- Camilleros y afanadores
- Técnicos y personal de laboratorio
- Asesoría a pacientes y terapia
- Limpieza
- Seguridad
- Comedor

2. Datos sociodemográficos:

- Nombre del participante:
- Sexo (masculino/femenino):
- Edad (años):
- Fecha de nacimiento: dd/mm/aaaa
- Estado civil (soltero/casado o unión libre/ viudo):

3. Nivel máximo de estudios:

- Menos que licenciatura
- Licenciatura o mayor

4. Turno laboral:

- Matutino
- Vespertino
- Nocturno
- Otro

5. Respecto a sus actividades de trabajo diarias. ¿Cómo es su contacto con pacientes?

Contacto nulo o limitado
Contacto alto

**6.**	**¿Se ha realizado PCR para detectar SARS-CoV-2?**<br><br>No<br>Sí, con resultado negativo<br>Sí, con resultado positivo<br>Si resultado positivo, fecha de la prueba: dd/mm/aaaa
**7.**	**¿Ha presentado síntomas o ha tenido la sospecha de haber tenido COVID-19?**<br><br>Sí /No
**8.**	**Desde marzo 2020 a la fecha: ¿Ha presentado alguna sintomatología relacionada con infección por SARS-CoV-2?**<br><br>Sí /No<br>En caso de que sí, ¿cuáles?<br>Mialgias-artralgias<br>Cefalea<br>Tos<br>Odinofagia<br>Rinorrea<br>Disnea<br>Fiebre<br>Diarrea<br>Conjuntivitis<br>Náusea<br>Escalofríos<br>Alteraciones del olfato<br>Disgeusia
**9.**	**¿Usted padece alguna enfermedad crónica (comorbilidad)?**<br><br>Sí /No
**10.**	**¿Se aplicó la vacuna de inmunización vs virus de influenza estacional la última temporada?**<br><br>Sí /No
**11.**	**¿Tiene usted algún familiar que haya sido diagnosticado con SARS-CoV-2?**<br><br>Sí /No

**12.**	**¿Con cuántas personas vive usted?**
**13.**	**¿Vive con personas que se han mantenido laborando fuera de casa durante la pandemia?**<br><br>Sí /No
**14.**	**¿Utiliza transporte público para llegar o regresar de su lugar de trabajo?**<br><br>Sí /No
**15.**	**Respecto a sus actividades de trabajo diarias, ¿usted atiende o ha atendido a personas positivas a COVID-19?**<br><br>Sí /No
**16.**	**¿Cuál es la forma en que actualmente usted está trabajando?**<br><br>Sólo en el Instituto Nacional de Rehabilitación<br>Además en otra institución No-Covid<br>Además en otra institución Covid
**17.**	**¿Usted conoce y utiliza el equipo de protección personal específico?**<br><br>Sí /No
**18.**	**¿Cuántas comidas realiza al día?**<br><br>Menos de tres comidas<br>Tres comidas o más
**19.**	**¿Realiza ejercicio o alguna actividad física?**<br><br>Sí /No
**20.**	**¿Cuántas horas en promedio duerme al día?**
