# Supplementary material for: Serological prevalence of SARS-CoV-2 infection and associated factors in healthcare workers in a “non-COVID” hospital in Mexico City
Source: PLoS One. 2021 Aug 12;16(8):e0255916. doi: 10.1371/journal.pone.0255916 (PMC8360585; doi:10.1371/journal.pone.0255916)
Supplement: S2 File — (PDF) [file pone.0255916.s008.pdf]

Questionnaire for the study: Serological prevalence of SARS-CoV-2 infection and associated factors in healthcare workers in a “non-COVID” hospital in Mexico City.
Type of questionnaire: Direct, face to face interview.

1. **Work group:**<br><br>• Administrative<br>• Scientific research<br>• Medical personnel<br>• Nursing<br>• Stretcher-bearers and orderlies<br>• Technicians and lab personnel<br>• Therapists and patient counseling<br>• Janitorial<br>• Security<br>• Food services
2. **Sociodemographic data:**<br><br>• Participant name:<br>• Sex (male/female):<br>• Age (years):<br>• Date of birth: dd/mm/yyyy<br>• Marital status (single / married or civil union / widow/er):
3. **Educational level:**<br><br>• Less than University bachelor’s degree<br>• University bachelor’s degree or higher
4. **Work shift:**<br><br>• Morning<br>• Evening<br>• Night<br>• Other
5. **Regarding your daily work activities, what is your degree of contact with patients?**<br><br>No or limited contact<br>High contact

**6.**	**Have you had an RT-PCR test to detect SARS-CoV-2?**<br><br>No<br>Yes, with negative result<br>Yes, with positive result<br>If positive result, date of test: dd/mm/yyyy
**7.**	**Have you resented symptoms or suspect having had COVID-19?**<br><br>Yes / No
**8.**	**Since March 2020 until now, have you presented any symptoms related to a SARS-CoV-2 infection?**<br><br>Yes / No<br>If yes, which symptoms?<br>Muscle and joint pain<br>Headache<br>Cough<br>Odynophagia<br>Rhinorrhea<br>Dyspnea<br>Fever<br>Diarrhea<br>Conjunctivitis<br>Nausea<br>Shivering<br>Olfactory alterations<br>Dysgeusia
**9.**	**Do you have any chronic disease (comorbidity)?**<br><br>Yes / No
**10.**	**Did you get the seasonal flu vaccine the last season?**<br><br>Yes / No
**11.**	**Do you have a family member that was diagnosed with SARS-CoV-2 infection?**<br><br>Yes / No
**12.**	**With how many people do you live?**

**13.**	**Do you live with people that have worked outside the home during the pandemic?**<br><br>Yes / No
**14.**	**Do you use public transportation for your work commute?**<br><br>Yes / No
**15.**	**Regarding your daily work activities, do you attend to COVID-19 patients?**<br><br>Yes / No
**16.**	**How are you currently working?**<br><br>Only in the Instituto Nacional de Rehabilitación<br>Additionally in another non-Covid institution<br>Additionally in another Covid institution
**17.**	**Are you familiar with and do you use personal protection equipment?**<br><br>Yes / No
**18.**	**How many meals do you eat per day?**<br><br>Less than three meals<br>Three or more meals
**19.**	**Do you practice exercise or another physical activity?**<br><br>Yes / No
**20.**	**How many hours per day do you sleep on average?**
